# Efficacy and safety of universal (TCRKO) ARI-0001 CAR-T cells for the treatment of B-cell lymphoma

**DOI:** 10.3389/fimmu.2022.1011858

**Published:** 2022-10-06

**Authors:** Noelia Maldonado-Pérez, María Tristán-Manzano, Pedro Justicia-Lirio, Elena Martínez-Planes, Pilar Muñoz, Kristina Pavlovic, Marina Cortijo-Gutiérrez, Carlos Blanco-Benítez, María Castella, Manel Juan, Mathias Wenes, Pedro Romero, Francisco J. Molina-Estévez, Concepción Marañón, Concha Herrera, Karim Benabdellah, Francisco Martin

**Affiliations:** ^1^ Department of Genomic Medicine, Pfizer-University of Granada-Andalusian Regional Government Centre for Genomics and Oncological Research (GENYO), PTS, Granada, Spain; ^2^ LentiStem Biotech, Pfizer-University of Granada-Junta de Andalucía Centre for Genomics and Oncological Research (GENYO), PTS, Granada, Spain; ^3^ Department of Celular Biology, Faculty of Sciences, University of Granada, Granada, Spain; ^4^ Cellular Therapy Unit, Maimonides Institute of Biomedical Research in Córdoba (IMIBIC), Reina Sofia University Hospital, University of Córdoba, Córdoba, Spain; ^5^ Department of Hematology, ICMHO, Hospital Clínic de Barcelona, Barcelona, Spain; ^6^ Department of Oncology, University of Lausanne, Épalinges, Switzerland; ^7^ Department of Biochemistry and Molecular Biology III and Immunology, Faculty of Medicine, University of Granada, Granada, Spain

**Keywords:** CAR-T cells, lymphoma, TCRKO, CRISPR/Cas9, off-the-shelf, safety, large deletions

## Abstract

Autologous T cells expressing the Chimeric Antigen Receptor (CAR) have been approved as advanced therapy medicinal products (ATMPs) against several hematological malignancies. However, the generation of patient-specific CAR-T products delays treatment and precludes standardization. Allogeneic *off-the-shelf* CAR-T cells are an alternative to simplify this complex and time-consuming process. Here we investigated safety and efficacy of knocking out the TCR molecule in ARI-0001 CAR-T cells, a second generation αCD19 CAR approved by the Spanish Agency of Medicines and Medical Devices (AEMPS) under the Hospital Exemption for treatment of patients older than 25 years with Relapsed/Refractory acute B cell lymphoblastic leukemia (B-ALL). We first analyzed the efficacy and safety issues that arise during disruption of the TCR gene using CRISPR/Cas9. We have shown that edition of *TRAC* locus in T cells using CRISPR as ribonuleorproteins allows a highly efficient TCR disruption (over 80%) without significant alterations on T cells phenotype and with an increased percentage of energetic mitochondria. However, we also found that efficient TCRKO can lead to on-target large and medium size deletions, indicating a potential safety risk of this procedure that needs monitoring. Importantly, TCR edition of ARI-0001 efficiently prevented allogeneic responses and did not detectably alter their phenotype, while maintaining a similar anti-tumor activity *ex vivo* and *in vivo* compared to unedited ARI-0001 CAR-T cells. In summary, we showed here that, although there are still some risks of genotoxicity due to genome editing, disruption of the TCR is a feasible strategy for the generation of functional allogeneic ARI-0001 CAR-T cells. We propose to further validate this protocol for the treatment of patients that do not fit the requirements for standard autologous CAR-T cells administration.

## Introduction

Genetically modified T cells hold great promise for the treatment of cancer, among them, chimeric antigen receptor (CAR) engineered T cells have been particularly successful for the treatment of hematological cancers (1). Treatment with anti-CD19 CAR-T cells achieved durable responses in patients with relapsed and/or refractory (R/R) acute lymphoblastic leukemia (ALL), chronic lymphocytic leukemia (CLL) and diffuse large B cell lymphoma (DLBCL) (2–6). This remarkable therapeutic activity has resulted in FDA/EMA approvals for six ATMPs up-to-date: *Kymriah (tisagenlecleucel)* for ALL, DLBCL and Follicular lymphoma; *Yescarta (axicabtageneciloleucel)* for DLBCL and primary mediastinal B cell lymphoma, *Tecartus (brexucabtageneautoleucel)* for mantle cell lymphoma (MCL) and ALL, *Breyanzi (lisocabtagenemaraleucel)* for DLBCL, *Abecma (idecabtagenevicleucel)* and *Carvykti (ciltacabtagenautoleucel)* for multiple myeloma (MM) (National Cancer Institute). In Spain, the ARI-0001 CAR-T cell therapy (in phase 2 study, NCT04778579), developed at Hospital Clínic of Barcelona, has been approved for ‘hospital exemption’ (HE) by the AEMPS for B-ALL patients older than 25 years. In addition, ARI-0001 (varnimcabtagene-autoleucel) has recently obtained the PRIority MEdicine (PRIME) designation from the EMA (7).

In spite of the outstanding clinical benefit of CAR-T cells, there are still important challenges associated with the manufacturing process since this therapy relies on autologous T cells (8–10). Most of these limitations can be overcome by manufacturing CAR-T cells using T cells from healthy donors. However, allogeneic cells can recognize mismatched major and minor histocompatibility antigens of patient cells leading to off-tumour graft-versus host disease (GvHD). Although, a naturally no alloreactive subsets of innate-like T cells such us γδT cells, can be used, these cells subset compromise only a 0.5% to 10% of peripheral blood mononuclear cells. Alternatively, genome editing strategies are the most promising approaches to make universal third-party CAR-T cells that can be used *off-the-shelf*, addressing the previous issues and avoiding graft-versus-host-disease (GvHD). The alpha beta T cell receptor (αβTCR) constitutes the optimal target to generate universal T cells, owing to its determining role in alloreactivity (11–16). Since the T cell receptor constant α chain (*TRAC*) expression was first eliminated by Torikai et al. using zinc finger nucleases (ZFNs) (17), multiple preclinical studies have been conducted targeting one or several genes though multiple gene-editing strategies (10, 15, 18–23). In 2017, the first clinical trial using genome edited CAR-T cells was approved. The authors eliminated the T cell receptor (TCR) expression using Transcription activator-like effector nuclease (TALEN) technology and treated paediatric and adult patients with R/R B-ALL. An overall survival of 55% was reported on patients that did not fit the requirement for autologous CAR-T cell therapies (24). Today, different allogeneic CAR-T products, mainly generated with TALEN and CRISPR/Cas9 tools, are currently undergoing Phase 1/2 clinical trials, reporting very promising responses (25–28).

However, these next-generation products are not risk-free. Apart from the *off-target* cleavage potential, other unwanted effects can occur, including translocations in cases of simultaneous DNA breaks or *on-target* mutagenesis. DNA double strand breaks (DSBs) generated after genome editing are mainly repaired by non-homologous end joining pathway (NHEJ) and homologous recombination (HR) (29); other mechanisms are alternative end joining (Alt-EJ), also known as microhomology-mediated end joining (MMEJ); and single-strand annealing (SSA) (30). Those different mechanisms create deletions and insertions of variable sizes (indels). Typically, indels are small (<50pb), although larger deletions (from hundreds of base pairs to several kilobases) have been also detected after CRIPSR/Cas9 cleavage (31–34). These large deletions are difficult to predict, as they are only partially dependent on sequence (35, 36).

Here, we report an in depth analysis of the feasibility of generating safe and efficient universal TCRKO ARI-0001 cells for the potential treatment of patients that do not fit the requirements for standard autologous CAR-T cells administration. We have shown that *TRAC* disruption in T cells using CRISPR as ribonucleoproteins (RNPs) enables a highly efficient TCR disruption (over 80%) without significant alterations on T cells. However, we also found that this procedure may promote *on-target* large deletions, indicating a potential safety risk that needs monitoring. Importantly, TCRKO ARI-0001 cells did not cause alloreactivity, maintained their phenotype and had a similar *ex vivo* and *in vivo* anti-tumor activity compared to unedited ARI-0001 CAR-T cells.

## Materials and methods

### Cells

Aliquots of apheresis products from healthy donors were obtained under informed consent at Reina Sofia´s Hospital (Córdoba, Spain). Peripheral blood mononuclear cells (PBMCs) were collected by Ficoll gradient (Lymphosep, Biowest) and frozen. PBMCs were thawed and cultivated in TexMACs medium (Miltenyi Biotec) supplemented with 20ng/ml IL-2 (Miltenyi Biotec) or 10ng/ml IL-7 and 10ng/ml IL-15 (Miltenyi Biotec), 5% human AB serum and 1% Penicillin/Streptomycin (P/S, Biowest) at a density of 10^6^ cells/ml, and maintained at 37°C, 5% CO2. The medium was refreshed every 48h. One day after thawing, cells were activated with TransAct T Cell (Miltenyi Biotec). To compare different media and activation protocols, we also used X-vivo 15 (Lonza) and Dynabeads (ThermoFisher).

Namalwa (ATCC^®^ CRL-1432) and Jurkat (ATCC^®^ TIB428 152) cells were expanded in RPMI 1640 Medium (Biowest) supplemented with 10% Fetal Bovine Serum (FBS, Gibco) and 1% P/S (Biowest) at 37°C in a 5% CO_2_ humidified incubator. HEK-293T (ATCC^®^ CRL-11268) cells were maintained in DMEM (Biowest) supplemented with 10% FBS (Gibco) and 1% P/S (Biowest) at 37°C and 10% CO_2_ atmosphere. Namalwa and Namalwa CD19KO cells expressing enhanced GFP (eGFP) and Nanoluciferase (NanoLuc) were previously generated in the laboratory (37). All the cell lines were tested for mycoplasma contamination using the MycoAlert Mycoplasma Detection Kit (Lonza).

### T cell electroporation and lentiviral transduction

Primary human T cells stimulated with TransAct T Cell Reagent (Miltenyi Biotec) and after 48h were transduced with the lentiviral particles at the desired multiplicity of infection (MOI) through spinoculation (800 x g for 60 min at 32°C). The medium was refreshed after 5 hours of incubation. After two/three days T cells were incubated with RNP composed of recombinant Cas9 (IDT) and a guided RNA (gRNA_TRAC_: ucaggguucuggauaucugu) against the first exon of the constant chain of the *TRAC* (GenScript) assembled in a molar ratio 3:1. The mix was electroporated using the 4D-Electroporator (Lonza), following manufacturer´s instructions. After electroporation, cells were diluted with culture medium and expanded as described above. Seven days after transduction, the percentage of edited and transduced cells, together with the phenotypic characterization, were determined by flow cytometry. The TCR edition efficiency was also corroborated by sequencing (see below).

### Lentiviral vector production and titration

For lentiviral vector generation, HEK-293T cells were seeded in 10 cm plates (Sarsted, Newton, NC) with a confluence lower than 90% and transfected using a second-generation system based on three plasmids: (1) Plasmid-vector (ARI-0001), (2) HIV virus packaging plasmid (pCMVΔR8.9) (http://www.addgene.org/Didier_Trono), and (3) VSV-G envelope plasmid (pMD2.G) (http://www.addgene.org/Didier_Trono), in a 10:7:3 ratio and using as transfection reagent polyethylenimine (PEI, Alfa Aesar, prepared in-house). Viral supernatants were collected at 30, 48 and 72h after transfection, filtered through a 0.45 μm filter (Nalgene, Rochester, NY) and concentrated by ultracentrifugation using the SW32 Ti rotor (Beckman) at 23000 rpm for 2h at 4°C. Finally, the LVs were resuspended in TexMACs, aliquoted, and stored at -80°C. Viral titers were determined by transducing easy-to-transduce cells (Jurkat cells) with serial dilutions of viral particles for 5 hours. After, 3-4 days, the percentage of positive cells was determined by FACS and transducing units per ml (TU/ml) were estimated according to the formula: [(10^5^ plated cells × %positive cells/100]/ml of viral supernatant.

### Mutagenesis analysis

#### On target analysis

Genomic DNA (gDNA) was extracted from pools of WT and edited cells using the QIAamp genomic DNA kit (Qiagen). Amplification was performed using KAPA2G Fast Hot Start Ready Mix (Sigma-Aldrich). Each PCR reaction was carried out in a final volume of 50µl, using 10ng of gDNA as template using the pair-1 primers (**Table S1**). All samples were run into duplicates in a thermocycler (Veriti, ThermoFisher) with the programme: 95°C for 5 min, followed by 40 cycles of 45 sec at 94°C, annealing at 60°C for 30 sec and extension at 72°C for 30 sec, and a final extension of 10 min. For downstream Sanger sequencing, PCR products were purified using the QIAquick PCR Purification Kit (Qiagen). Sequences were analyzed using ICE software (https://ice.synthego.com/) to determine knockout score and indel distribution.

#### Off-target analysis

Cas-OFFinder web tool was employed for off-target prediction of designed gRNA_TRAC_, (http://www.rgenome.net/cas-offinder/). Six potential targeted positions were amplified through PCR reaction, and then analyzed by Sanger sequencing. The percentage of edition was determined using Interference of CRISPR Edits (ICE) software (https://ice.synthego.com/). Primers used are listed in **Supplementary Table 1**.

#### Deep sequencing of *TRAC* locus in CRISPR/Cas9 edited cells by Illumina sequencing

Two different amplicons of 452 bp (pair-1, **Table S1**) and 532 bp (pair-4, **Table S1**) covering the gRNA_TRAC_ cutting site were amplified under the same conditions described previously. Primers are listed in **Supplementary Table 1**. Amplicons were paired-end sequenced using MiSeq™ (Illumina). Adapters were trimmed using ‘Cutadapt’ [https://doi.org/10.14806/ej.17.1.200] for universal Illumina primers and quality control was performed using fastqc [http://www.bioinformatics.babraham.ac.uk/projects/fastqc]. Alignment to the reference genome (UCSC) was performed using bwamem. Aligned sequences were converted into BAM format file, sorted and indexed using Samtools (38) so they could be visualized using IGV (39). Indel frequency was obtained using Cas-Analyzer online tool (CRISPR RGEN Tools, http://www.rgenome.net/cas-analyzer/#!result) (40).

#### PCR analysis of large deletions

A 4kb region surrounding the cut site was amplified using Fw8 and Rv8 primer set (pair-8, **Table S1**) and 50ng gDNA as a template. Samples were run into duplicate in Verity system using the program: 95°C for 3 min, followed by 40 cycles of 15seg at 94°C, annealing at 62°C for 2.5 minand extension at 72°C for 3 min, and a final extension of 1 min. After amplification, PCR products were run in a 1% agarose gel with a 1kb ladder (ThermoFisher) and visualized using Image Lab software (Bio-Rad).

#### Isolation and characterization of large deletions

A fraction of gel containing different size amplicons was extracted using QIAquick Gel Extraction Kit (Qiagen) and cloned into a PCR2.1 plasmid. Competent bacteria were transformed and seeded on a LB agar plates containing X-Gal/IPTG (to allow the blue and white screening colonies) and 50µg/mL of ampicillin. After 37°C overnight incubation, 40 white colonies containing the inserts were resuspended in water and theinserts size analyzed by PCR. For downstream sequencing, plasmid DNA was extracted using Plasmid DNA Miniprep Kit (Quiagen) from 20 different clones. All sequences obtained were aligned to the reference genome (hg38) using UCSC BLAT (41) and visualized using IGV (39). Presence of repetitive elements was evaluated using UCSC RepeatMasker (42). Once aligned, split sequences allowed the identification of large deletions. Microhomologies were analyzed using the Biostring package and an R script developed by Owens *et al.* (35). This customized script uses the 5’ and 3’ 10 bp of both up and downstream breakpoints in each deletion to identify a microhomology.

### Flow cytometry

CAR19 expression was detected with a biotin-conjugated goat anti-mouse Fab IgG (Jackson Immunoresearch) and APC-conjugated streptavidin (ThermoFisher). For T cell characterization the following surface mAbs were used: PE-Cy7/PerCP-Cy5.5-hCD62L, PE/FITC-hCD45RA, PerCP-Cy5/APC-780-hCD3, PerCP-Cy5/APC-780-hCD2, APC-hCD25, UV-450/PeCy7-hCD4, UV-450/PeCy7-hCD8, APC-Cy7-hTIM-3, PE/eFluor506-hLAG3, APC/PE-hPD1, FITC-hCD39, Pacific Blue-hCCR7 and PE-Cy7-hCD28 from eBioscience (ThermoFisher). T cell subpopulations were defined as follows: Tnaive/Tstem cell memory (T_N_/T_SCM_): CD45RA+CD62L+; T central memory (T_CM_): CD45RA-CD62L+; T effector memory (T_EM_): CD45RA-CD62L- and T effector (T_EFF_): CD45RA+CD62L-. Staining was performed in the dark at 4°C for 20min. To analyze mitochondrial fitness cells were stained with Tetramethylrhodamine methyl ester (TMRM) and MitoTracker Green (MG) (ThermoFisher) at 37°C for 20 min in RPMI without phenol red (ThermoFisher). For mouse samples, prior to stain, the Fcγ receptors were blocked using anti-murine CD16/CD32 (ThermoFisher), FcR blocking (Miltenyi) and 5% mouse serum (Sigma Aldrich) on ice for 20 min. Cells were washed with 2ml of FACs buffer (PBS+3% BSA+2mM EDTA). Samples were acquired on a FACS CantoII or FACSDiva (BD Biosciences) cytometer, and data were analyzed by the FlowJo software (TreeStar). Total cell counts were determined using CountBright™ Absolute Counting Beads (ThermoFisher).

### Alloresponse assay

To evaluate the response of TCR edited T cells to alloantigens, CD3- and CD3+ T cells, washed and maintained 24h before the experiment in TexMACs medium without any cytokines supplement. T cells were labelled with Cell Trace Violet (CTV, ThermoFisher) following manufacturer´s instructions and cocultured with CD19 depleted-PBMCs from the same or a different donor at 1:5 Effector:target (E:T) ratio. No significant differences were observed in the PBMC number in the different allo-PBMCs. After six days, T cells were stained to detect CAR, CD3, CD62L, CD45RA CD25 and CD8. Proliferation (loss of CTV staining) was measured over CD3+CD8+CAR+ cells.

### Cytotoxic assay

Cytotoxic activity of αCD19 CAR-T cells was determined as previously described (37). Briefly, TCR^+^ or TCR^-^ CAR+ T cells (effectors) and CD19+ (target) or CD19- (non-target) eGFP-NanoLuc Namalwa cells were co-cultured into duplicated at the indicated E:T ratios in non-supplemented TexMACs at a concentration of 2 x 10^4^ target cells per well. After 48h, the total counts of Namalwa and effector cells were determined using Count Bright™ Absolute Counting Beads (ThermoFisher). T cell phenotype and exhaustion markers were determined by flow cytometry. Specific lysis was determined using the formula:


$$Specific\;lysis=(1-\frac{\frac{\%CD19+in\;CAR+}{\%CD19-in\;CAR+}}{\frac{\%CD19+in\;NT}{\%CD19+in\;NT}})\times 100$$

Supernatants were collected from 24h co-cultures and frozen at -80°C. TNF-α and IFN-γ were measured with ELISA MAX Deluxe Set (Biolegend, San Diego, California, USA) following manufacturer’s instructions.

For the re-challenge assay, we seeded a co-culture of CAR-T cells and Namalwa cells at ratio 1:2 or 1:1 into 8 identical wells. Every 48h or 24h, one well was used for FACS analysis and, in the other replicates, half of the volume was replaced with the same initial number of Namalwa cells. The assay ended when the percentage of tumoral cells was superior to 20%.

### *In vivo* animal models and bioluminescence analysis

All experiments were performed according to a protocol approved by the Institutional Animal Care and Use Committee of the University of Granada (procedures 92-CEEA-OH2015) and were in concordance with the European Convention for the Protection of Vertebrate Animals used for Experimental and Other Scientific Purposes (CETS # 123) and the Spanish law (R.D. 53/2013).

Six to eight-week-old NOD/scid-IL-2Rnull (NSG, The Jackson Laboratory) mice were inoculated intravenously (i.v.) with 0.3x10^6^ GFP-NLuc Namalwa cells per mice, three days later the mice were i.v. infused with thawed CAR T cells (50%CAR+, 2x10^6^ CAR T cells/mice), non-transduced T cells (NT, 4x10^6^) or vehicle (PBS). Tumor tracking was assessed twice a week by bioluminescence imaging (BLI). For bioluminescence analysis, furimazine (Nano-Glo, Promega) was dissolved at 1/60 in PBS and injected intraperitoneally prior acquisition on an IVIS Spectrum *In Vivo* Imaging System (PerkinElmer) during 180s, open field. The images were analyzed using the Living Image 3.2 (PerkinElmer) program and AURA Imaging Software 3.2 (Spectral Instruments Imaging). Mice were monitored for signs of GvHD such as weight loss, reduced mobility, hunched posture, fur loss, tachypnea. After 1 month, a re-challenge was conducted by re-inoculating intravenously a new dose of GFP-NLucNamalwa cells. Mice were euthanized after experiencing a weight loss greater than 20% or when BLI reached values above 5x10^7^ Photons/sec. After the sacrifice of the mice, samples of blood, brain, bone marrow, liver and spleen were analyzed for Namalwa cell and human T/CAR-T cell presence by FACS. Cells were obtained by mechanical disruption of the liver and spleen, by perfusion of the femur and tibias and by mechanical disruption followed by Percoll gradient separation of brain. Blood was extracted from aortic vein and diluted in EDTA.

#### Extracellular flux analysis

Mitochondrial function of T cells and CAR-T cells were assessed 10 days after initial stimulation with a-CD3/CD28 using Agilent Seahorse XF HS Mini Analyzer and T cell metabolic profiling kit (Agilent Technologies). CAR-T cells (WT or TCRKO) were suspended in XF RPMI medium containing 10mM glucose, 2 mM L-glutamine, and 1 mM sodium pyruvate then seeded at 1 x 10^5^ cells per well in XFp PDL miniplates. “Resting” cells were maintained at 37°C in a non-CO_2_ incubator during instrument calibration. Cellular oxygen consumption rates (OCRs) and extracellular acidification rates (ECARs) were measured under basal conditions and following treatment with 1.5µM oligomycin A, 2.5µM BAM15, and 0.5µM rotenone with 0.5µM antimycin A (Agilent Technologies). Data were analyzed using Seahorse Analytics software (https://seahorseanalytics.agilent.com/).

### Data analysis

Statistical analyses were performed using GraphPad Prism 9 software (GraphPad Software Inc., La Jolla, CA, USA). The performed statistical test is indicated in every figure legend. Data were expressed as the mean ± standard error of the mean (SEM). Survival curves were obtained using the Kaplan–Meier analysis.

## Results

### Highly efficient CRISPR-mediated edition of the TRAC locus results in disruption of TCR expression with conserved T cell physiology

**Figure 1:**
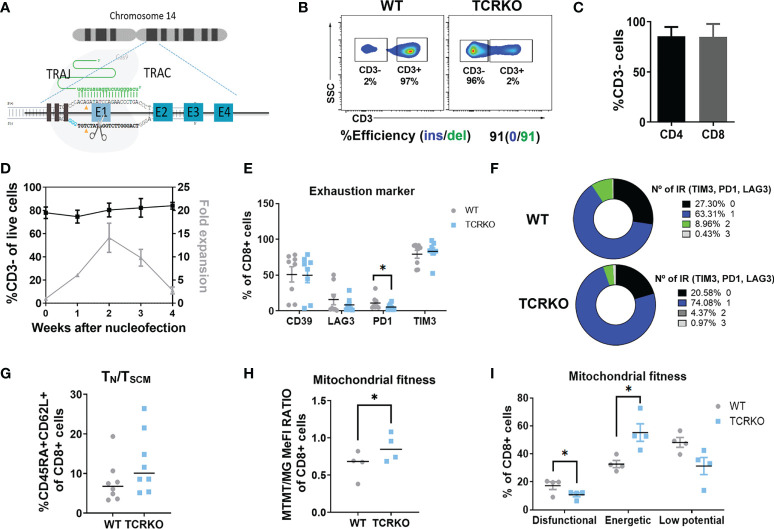
Generation of TCR negative T cells by Cas9/gRNA_TCRA_ RNP electroporation. **(A)** Diagram of CRISPR/Cas9 system composed by a specific gRNA against exon 1 (RNP_TRAC_) of the *TRAC* locus and Cas9 recombinant protein. **(B)** Efficiency of TCR disruption in primary T cells at day 3 post-nucleofection. CD3 expression by flow cytometry (top plots) and ICE analysis of PCR product (bottom) are shown. **(C)** Graph showing % of CD3- in CD4+ and CD8+ T cell populations (4 independent donors, n=8). **(D)** Graph showing % of CD3- of edited T cells (left Y axis) and expansion over time (right Y axis) (4 independent donors, n=4). **(E)** Percentage of CD39+, LAG3+, PD1+ and TIM3+ of CD8+ mock electroporated (WT) and TCRKO T cells **(F)** Spice graphs showing the percentage of expression of 0(black), 1(blue), 2(green) and 3(gray) exhaustion markers/inhibitory receptors (IR) (TIM3, PD1 and LAG3) in CD8+ WT (left) and TCRKO (right) T cells **(G)** Percentage of T_N_/T_SCM_ CD8+ T cells, determined as CD45RA+CD62L+ (7 independent donors, n=8). **(H)** Quantification of flow cytometry data at day 10 post activation showing the ratio of mitochondrial membrane potential (MTRM) to mitochondrial mass (MG) in TCRKO and mock electroporated CD8+ T cells and **(I)** Percentage of dysfunctional(MG+TMRM-), energetic (MG+TMRM+) and low potential mitochondria(MG-TMRM-) in TCRKO and mock electroporated CD8+ T cells (WT) (4 independent donors, n=4).Statistics are based on paired, two-tailed Student´s t-test **(H, I)**, *p<0.05. Graphs show mean ± SEM.

Clinical translation of universal CAR-T cells requires highly efficient and safe protocols to generate allogeneic products. Nowadays, the gold standard method is the disruption of the TCR though genome editing to prevent GvHD. Based on the literature (43), we selected nucleofection with Cas9/gRNA RNP targeting the first exon of *TRAC* gene (RNP_TRAC_) (**Figure 1A**) as the method of choice. This method achieved consistent TCR disruption (TCRKO) of over 80% in primary human T cells from different healthy donors (**Figures 1B, C**). TCRKO efficiency was equivalent in both CD4+ and CD8+ T cells (**Figure 1C**) and was stable over time in culture keeping their proliferative capacity up to four weeks (**Figure 1D**).The percentages of CD8+ cells expressing memory markers (**Figure S1A**), exhaustion markers/inhibitory receptors (**Figures 1E, F**), and the levels of T_N_/T_SCM_ (**Figure 1G**) and T_CM_ (**Figure S1B**. See **Figure S1C** for gating strategy), were maintained similar to those in unedited (WT) T cells. We also investigated the potential side effects of TCRKO on mitochondrial fitness by measuring dysfunctional, energetic, and low potential mitochondria in CD8 population (see M&M for details). Interestingly, deletion of the TCR increased the percentage of energetic mitochondria, while reduced the percentage of dysfunctional mitochondria (**Figures 1H, I**). Altogether these data indicate that TCRKO T cells do not present a major functional disadvantage compared to WT T cells in the settings used to culture T cells *in vitro*.

### Genome editing at early days after T cell isolation renders high efficiencies of gene disruption without compromising safety

In order to reduce expansion time and to maintain T cell phenotypes, genome editing of T cells should be done shortly after their isolation from the patient. However, freshly isolated T cells might be more susceptible to undesired genotoxicities compared to expanded (more differentiated) T cells as consequence of it more stem-like genome (44, 45). We therefore edited T cells from healthy donors at day 4 or day 17 after their isolation. We first analyzed potential differences in the frequency of off-target indels. Using primary T cells edited with similar efficiency (around 90% TCRKO), we showed by ICE analysis that none of the top 6 predicted off-target sites generated detectable cleavage at day 4 nor day 17 (**Figure S2A**).

**Figure 2:**
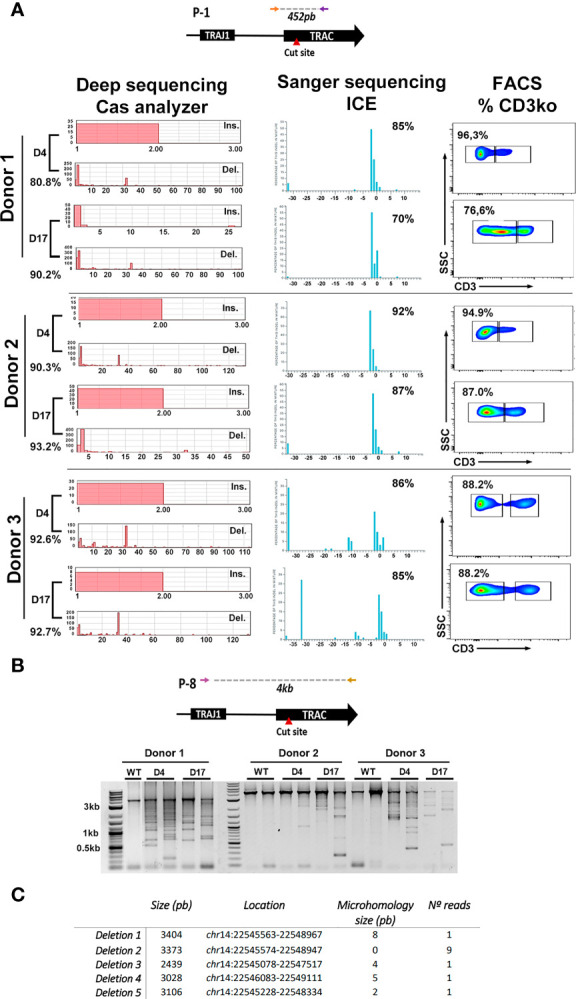
Indel analysis of CRISPR/Cas9 treated T cells. **(A)** Top, scheme of the PCR product analyzed after *TRAC* genome editing using Cas9/RNP_TRAC_ RNPs. Bottom, analysis of the genome editing efficacy using deep sequencing and Cas analyzer (left), Sanger sequencing and ICE analysis (Middle) and flow cytometry (Righ). TCR genome editing was performed in primary T cells from three different donors (indicated at the left) at day 4 and day 17 after isolation. Left: Indel frequency obtained by Cas analyzer are shown for each day (left-bottom in each day). Insertions (Ins.) and deletions (Del.) sizes are visualized as graphs. Middle: Indel frequency obtainned by Sanger sequencing and ICE are show at the top-right corner of each graph. Graphs also display the inferred insertion and deletion (indel) distribution. Each bar shows the size of the insertion or deletion along with the percentage of genomes that contains it. Right: Flow cytometry dot-plots showing the percentage of TCR disruption, measured as the reduction of CD3 expression on edited cells. **(B)** Top: PCR primers used to detect large deletions at the *TRAC* locus located 2kb upstream and 2kb downstream the RNP target site. Bottom: Electrophoresis gel showing the different amplicons that arise after a PCR using the primers indicated to light up large deletions. PCRs were performed for three different donors at day 4 and day 17 as indicated. Non-edited T cells (WT) were used as controls for each donor. **(C)** Table showing the analysis of 13 different deletions found by bands isolation and Sanger sequencing. Deletion size, location and the presence of microhologies is indicated.

We next analyzed on-target indel distribution on primary T cells from three different donors that were edited at day 4 or day 17. Efficacy of genome editing on the different samples was determined by ICE (**Figure 2A**, middle graphs) and FACS analysis (percentage of TCRKO cells, measured determining the percentage of T cells that are CD3 negative) (**Figure 2A**, right plots). In addition, a more detailed indel distribution in the different samples was performed by deep sequencing of PCR amplicons followed by analysis using Cas-analyzer software (**Figure 2A**, left graphs and **Figure S2B, C**) (40). Independently of the system used to determine the indel distribution, we found that deletion of 2 (2nt) or 1 (1nt) nucleotides were the predominant indels in Donor 1 and donor 2, while a deletion of 32 (-32nt) was predominant in donor 3 (**Figure 2A** left-bottom and right-bottom graphs and **Figure S2C**). Interestingly, the -32nt deletion is under-represented in donors 1 and 2 (**Figure 2A** and **Figure S2C**), suggesting a donor-dependency of the type of indels generated, despite using identical RNP_TRAC_ and electroporation procedure.

Recent studies have shown a relative high presence of large deletions at CRISPR/Cas9 cut site. This phenomenon may be overstated in activated T cells submitted to TCR editing, (where TCR stimulation rapidly induces chromatin remodeling). To either rule out or to confirm this possibility we investigated the presence of large deletions on the TCR alpha constant region after CRISPR/Cas9 cleavage in primary T cells edited at different time points. A 4kb region surrounding the cut site was amplified from the gDNA of control (WT T cells) and T cells edited at day4 or day 17 (**Figure 2B**, top). As expected, WT samples showed a unique band of 4kb (that can present different intensities due to experimental variations) corresponding with the expected amplicon. However, the edited samples showed, in addition to this 4kb band, the presence of large deletions in the TCR locus in the three donors at both days of edition (**Figure 2B**, bottom). To isolate specific bands that correspond with different deletions, DNA was extracted from agarose gel and cloned into a PCR2.1 plasmid as described in M&M and 20 clones were analyzed (See M&M). A partial analysis of amplified bands revealed 5 distinct deletions that ranged from 2kb to 3kb likely generated by NHEJ and microhomology-mediated DNA repair (**Figure 2C** and **Figure S2D**).

Together, our results show no substantial differences in T cell edition time point regarding efficiency and safety. However, they also highlight the importance of monitoring small and large deletions on genome edited T cells, since they could become an important source of genomic instability

### TCR edited ARI-0001 CAR-T cells maintain their phenotype, mitochondrial fitness and their *in vitro* anti-tumor activity

**Figure 3:**
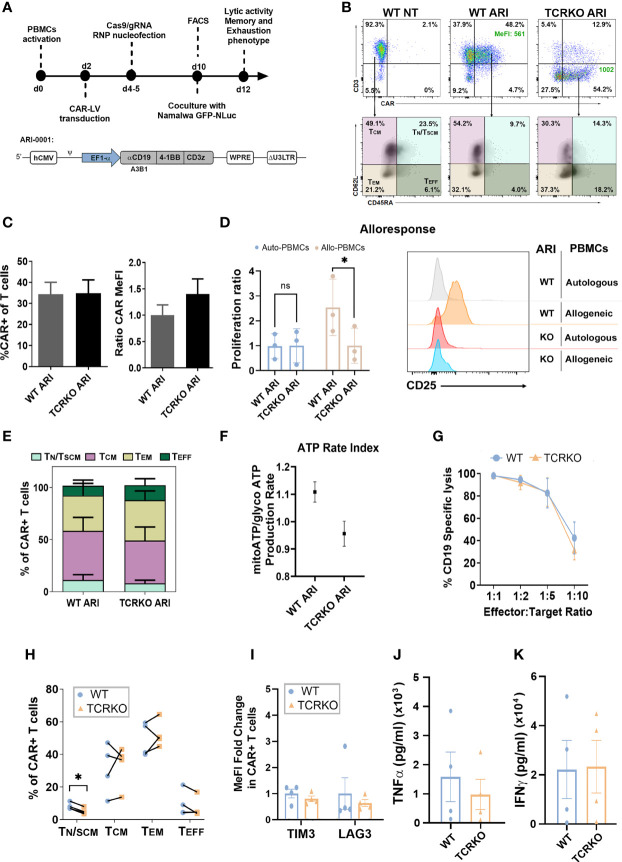
Generation of Universal α-CD19 CAR-T cells. **(A)** Top: Workflow of the experiments. Bottom: schematic representation of the ARI-0001-LV expressing α-CD19 CAR. **(B)** Top: Representative dot-plots showing CAR expression levels in control (WT NT, left), ARI-0001 (WT ARI, middle) and genome edited ARI-0001 (TCRKO ARI, right) T cells. Percentages (%) of CAR+ cells and expression levels (Median of Fluorescent Intensity – MeFI) are shown. Bottom: Dot-plots showing CD62L and CD45RA expression of control (left), ARI-0001 CAR (middle) and TCRKO ARI-0001 CAR (right) T cells. Percentages of T_N_/T_SCM_, T_CM_, T_EM_ and T_EFF_ are indicated. **(C)** Graphs showing the %CAR+ cells in TCRKO and WT CAR-T cells (left) and the relative CAR expression level (MeFI) related to WT ARI-0001 (right) (7 independent donors, n=11). **(D)** Alloresponse analysis. WT ARI-0001 and TCRKO ARI-0001 CAR T cells were labelled with Cell Trace Violet (CTV) and co-cultivated with allogeneic (allo) or autologous (auto) PBMCs. Graphs showing the proliferation ratio (left) and CD25 expression (right) of each CAR-T after 6 days of co-culture determined by FACS (3 independent donors, n=3). **(E)** Percentage of T_N_/T_SCM_, T_CM_, T_EM_ and T_EFF_ in non-stimulated TCRKO and WT ARI-0001 cells (4 independent donors, n=4). **(F)** Graph showing ATP rate index calculated by the ratio of mitochondrial ATP divided by glycolytic ATP. Donors = 2. Three replicates each. **(G)** Graph comparing specific lysis of CD19+ cells by WT and TCRKO CAR T cells at different effector:target (E:T) ratio after 48h of co-culture (4 independent donors, n=4). **(H)** Percentage of T_N_/T_SCM_, T_CM_, T_EM_ and T_EFF_ in TCRKO and WT ARI-0001 cells after 48h of co-culture with Namalwa cells at E:T ratio 1:2 (4 independent donors, n=4). **(I)** Fold change of TIM3 and LAG-3 expression levels of TCRKO ARI-0001 related to the expression in WT ARI-0001 cells after 48h of co-culture with Namalwa cells at ratio 1:2 (4 independent donors, n=4). **(J)** Secretion of human TNF-α and **(K)** IFN-γ from effector cells in response to target cells after 24 hours of co-culture (4 independent donors, n=4). Statistics are based on paired, two-tailed Student´s t-test **(D, H)**, *p<0.05. Graphs show mean ± SEM.

Once established the efficiency and potential risks of the procedure to disrupt the TCR, we next aimed at generating TCR edited (TCRKO) CAR T cells. We first compared different protocols using electroporation of RNPs and transduction with ARI-0001 LVs in different order and using different media for their transduction and expansion (**Figure S3**). Based on these studies, PBMCs from healthy donors (see M&M) were cultured in TexMACs medium supplemented with 20ng/ml IL2 and 5% human AB serum, activated with α-CD3/CD28 nanomatrix (T cell TransAct) and transduced with lentiviral vectors (LVs) expressing the ARI-0001 CAR (MOI=10). 2-3 days later, the cells were electroporated with CRISPR/Cas9/gRNA_TRAC_ RNPs to generate TCRKO ARI-0001 CAR-T cells. We maintained non-electroporated CAR T cells (WT ARI-0001) and non-transduced cells (NT) as control (**Figure 3A**). TCR-edited and non-edited T cells showed similar percentages of CAR expression (34.8% ± 21.1 vs 34.4% ± 18.8, p=0.86) although the expression levels (indicated by MeFI) were slightly higher in TCRKO CAR-T cells (**Figures 3B, C**, see **Figure S4A** for gating settings.). Of note, contrary to what was observed with WT ARI-0001 CAR-T cells, TCRKO ARI-0001 CAR-T cells (CD8+ and CD4+) proliferation (**Figure 3D**, left and **Figures S4B, C**) and activation (**Figure 3D**, right) was identical in the presence of allogeneic and autologous PBMCs. These data indicate that TCRKO ARI-0001 T cells do not present alloreactivity and may therefore be safe to be used in an allogeneic setting.

We next investigated the changes in T cell phenotype in unstimulated TCRKO ARI-0001 versus WT ARI-0001 CAR-T cells (10 days after initial stimulation with αCD3/αCD28). Despite donor variability, we observed a tendency for lower frequency of T_N_/T_SCM_ and T_CM_ in TCRKO ARI-0001 cells, although the differences did not reach significance (**Figure 3E**). In addition, WT ARI-0001 and TCRKO ARI-0001 cells had similar expression levels of co-inhibitory molecules such as TIM3 and LAG3 (**Figure S4D**). Since metabolic fitness is also related to memory-like T cells and the development of durable anti-tumor responses (46), we measured metabolic potential using Seahorse XF technology (47). These analyses showed equal metabolic fitness of WT- and TCRKO- ARI-0001 cells as indicated by similar ATP rate index (**Figure 3F**) and oxygen consumption rate, (**Figures S4E, F, G**).

Antitumoral efficacy of TCRKO ARI-0001 versus WT ARI-0001 CAR-T cells was evaluated using Namalwa cells, an aggressive Burkitt lymphoma-derived human cell model. Wild type (CD19+) or CD19KO Namalwa cells were seeded in presence of different number of WT and TCRKO ARI-0001 cells. In line with the minimal changes observed in terms of phenotype and mitochondrial activity between TCRKO and WT ARI-0001 CAR T cells, our analysis also showed no differences in their *in vitro* anti-tumor activity (**Figure 3G**, **Figure S4H**). Moreover, phenotypic analysis after tumor lysis showed a predominant population of T_EM_ in both conditions, although we observed a significant higher decrease of T_N_/T_SCM_ in TCRKO ARI-0001 cells compared with WT ARI-0001 cells (8% vs. 5%) (**Figure 3H**). In addition, WT ARI-0001 and TCRKO ARI-0001 CAR-T cells shared a comparable expression of LAG3 and TIM3 exhaustion markers after tumor cells killing (**Figure 3I**) as well as similar production of interferon (IFN)-γ and tumor necrosis factor (TNF)-α (**Figures 3J, K**).

**Figure 4:**
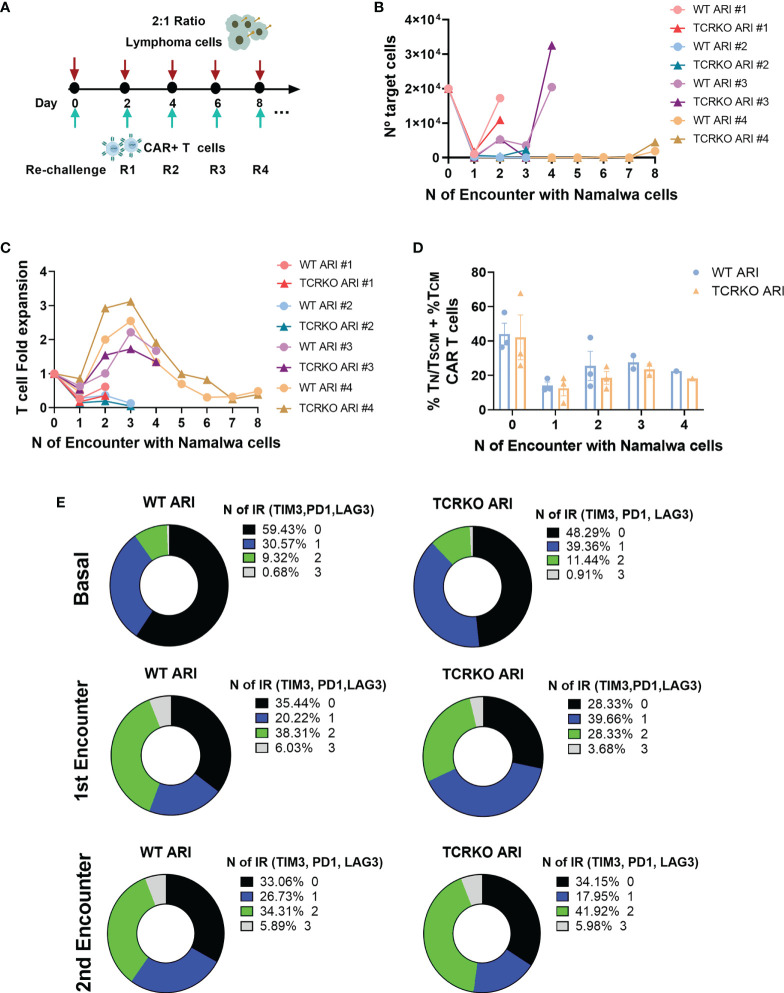
TCRKO CAR T cells maintain effector potency after repetitive tumor re-challenge. **(A)** Scheme of tumor re-challenge assay. CAR T cells were cocultured with Namalwa cells (E:T = 1:2) and rechallenged every two days. **(B)** Graph showing number of remaining tumor cells quantified every 48h. **(C)** T cell fold expansion after every encounter related to the initial numbers of T cells seeded. **(D)** Percentage of T_N_/T_SCM_ +T_CM_ of WT ARI-0001 and TCRKO ARI-0001 after the different challenges with Namalwa cells (4 independent donors, n=4). **(E)** Graphs showing the percentage of expression of 0(black), 1(blue), 2(green) and 3(gray) exhaustion markers/inhibitory receptors (IR) (TIM3, PD1 and LAG3) in WT (left) and TCRKO (right) ARI-0001 CAR-T cells before encounter (basal-top) and after one (1^st^ encounter-middle) and two encounters (2^nd^ encounter-bottom) with Namalwa cells (3 independent donors, n=3). Graphs show mean ± SEM.

The slight decrease in T_N_/T_SCM_ in the TCRKO CAR-T cells suggested that these cells could be less efficient than WT CAR-T cells if analyzed after repeated challenges with target cells. In order to study this possibility we performed the experiment shown in **Figure 4A**. However, TCRKO ARI-0001 CAR-T cells showed comparable lytic activity to WT ARI-0001 cells in a re-challenge assay (**Figures 4B, C**). In both CAR-T cells, subpopulations with increased persistence T_N_/T_SCM_ + T_CM_ dropped after the first encounter and increased after the second one (**Figure 4D**). As expected, the expression of 2 up to 3 inhibitor receptors (TIM3, PD1, LAG3) increased after the first encounter in both WT and TCRKO ARI-0001 cells, although this increment was higher after the second encounter in TCRKO ARI-0001 cells (**Figure 4E**, **Figures S5A, B, C**).

**Figure 5:**
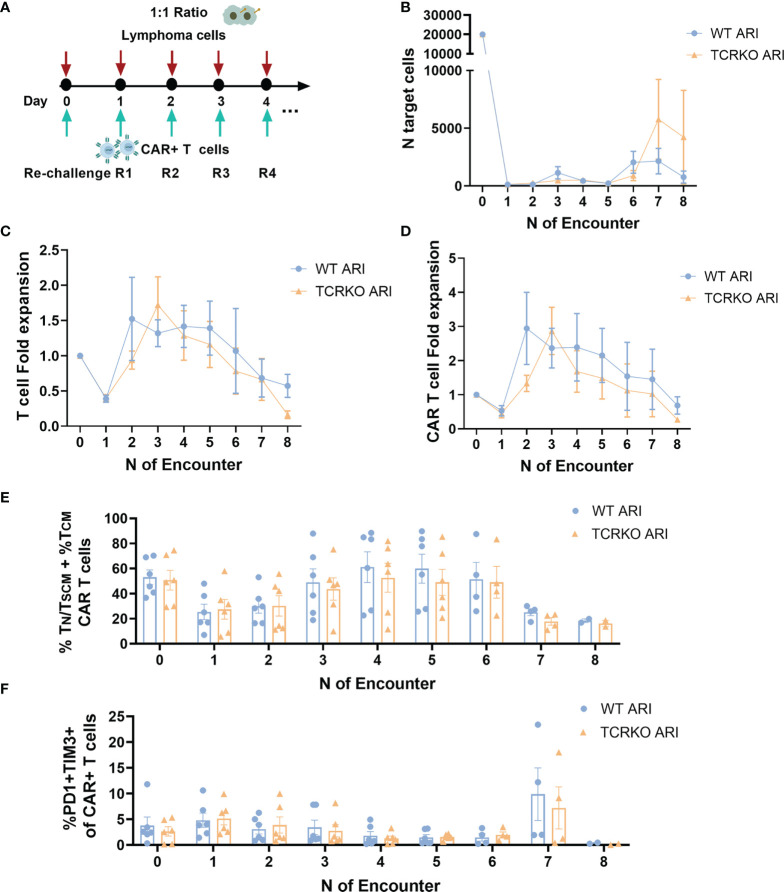
TCRKO CAR T cells maintain effector potency after highly repetitive tumor re-challenge. **(A)** Schema of second tumor re-challenge assay. CAR-T cells were co-cultured with Namalwa cells (E:T = 1:1) and rechallenged every day. **(B)** Graph showing number of remaining tumor cells quantified by flow cytometry every 24h. **(C, D)** Graph showing T cell **(C)** or CAR-T cells **(D)** fold expansion after every encounter relativized to the initial numbers of seeded cells. **(E)** Percentage of T_N_/T_SCM_ +T_CM_ of WT ARI-0001 and TCRKO ARI-0001 after the different re-challenges with Namalwa cells. **(F)** Graph showing the percentage of PD1+TIM3+ cells in WT ARI-0001 and TCRKO ARI-0001 after the different re-challenges with Namalwa cells. Graphs show mean ± SEM of n=6 independent donors/group.

To further stress the *in vitro* system, we set up a second tumor re-challenge assay in which CAR T cells were expanded for 10 days in the presence of IL-7 and IL15, co-cultured with Namalwa cells at E:T ratio of 1:1 and challenged every day (**Figure 5A**). As depicted in **Figure 5B**, both WT and TCRKO ARI-0001 cells mediated efficient killing of lymphoma cells after every re-challenge and achieved similar CAR T fold expansion (2.94 for WT ARI-0001 and 2.87 for TCRKO ARI-0001) as well as total T cell fold expansion (1.5 for WT ARI-0001 and 1.7 for TCRKO ARI-0001) (**Figures 5C, D**). Of note, both WT and TCRKO ARI-0001 cells acquired a predominant T_EM_ phenotype but with a significant percentage of T_N_/T_SCM_ + T_CM_ (T cells with increased potential for persistence) until the 6^th^ re-challenge (**Figure 5E**). In addition, both conditions displayed low co-expression of PD1 and TIM3 co-inhibitory molecules (**Figure 5F**). Since we aim to generate an *off-the-shelf* product, we also compared phenotype and antitumor efficacy of WT and TCRKO ARI-0001 CAR-T cells after a freeze-thaw cycle. Although we did not find any significant differences in lytic activity, frozen WT and TCRKO ARI-0001 cells presented a reduction in the percentage of T_N_/T_SCM_ + T_CM_ compared with fresh conditions. However, we did not find differences in the percentage of TIM3+LAG3+ between both populations (**Figures S5D, E, F**).

Altogether, our *in vitro* studies showed no significant differences between TCRKO ARI-0001 and WT ARI-0001 CAR T cells in terms of cytotoxicity, mitochondrial activity and T cell proliferation.

### CD19 CAR TCRKO T cells have similar anti-tumor activity and persistence to its WT counterpart in a Namalwa lymphoma mouse model

**Figure 6:**
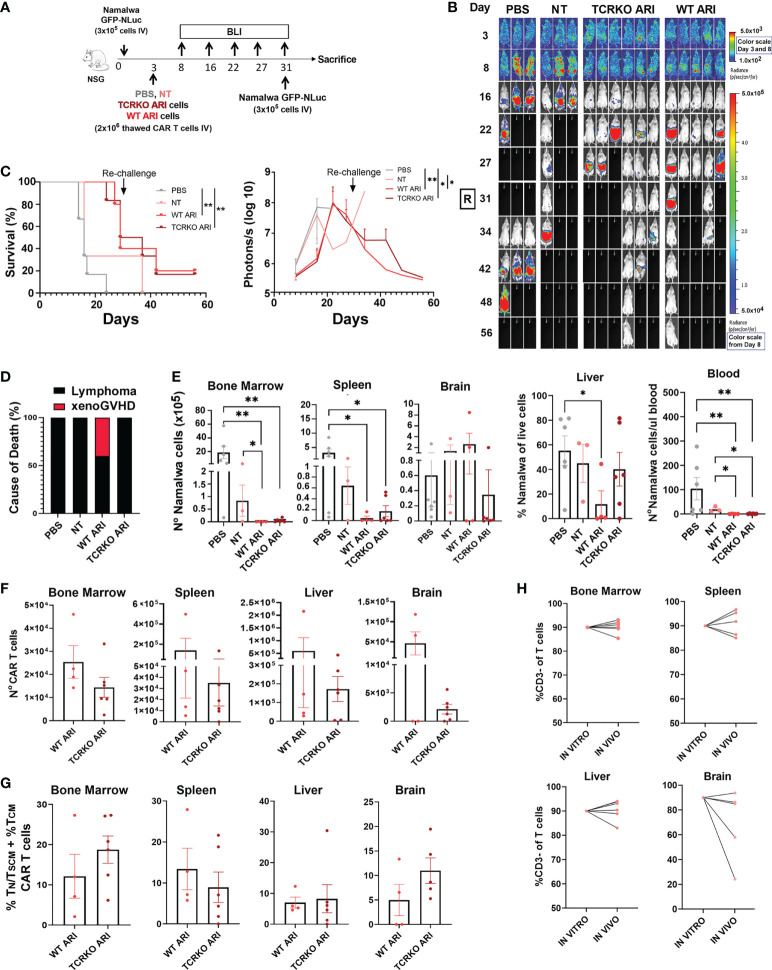
*In vivo* anti-tumor activity and phenotype of TCRKO ARI-0001 cells. **(A)** Schematic overview of the *in vivo* experimental design. **(B)** Representative BLI images of tumor burden at different days after Namalwa inoculation. A rechallange with new Namalwa cells was performed with alive mice at day 31. Color scale bar is shown for Day 3 and Day 8-56. Radiance scale is shown in each bar. **(C)** Left: Overall survival. Mice with > 20% body weight loss or high bioluminescence signal (>5x10^7^ Photons/sec) were sacrified (N=3 mice for untreated (PBS) and non-transduced (NT), N=5 mice for WT ARI-0001 and N=6 mice for TCRKO ARI-0001. CAR T cells were obtained from one healthy donor. Right: radiance quantitative trace of each group of mice represented as photons per second at the different days. **(D)** Incidence (%) of lymphoma- or xenoGVHD- related death in the different mouse groups. **(E)** Number of Namalwa cells in bone marrow, spleen, liver, brain and blood in NSG mice left untreated (PBS) or treated with NT, ARI-0001 CAR-T cells (WT ARI) and TCR-deficient ARI-0001 cells (TCRKO ARI) at the end point. **(F)** Number of CAR-T cells in bone marrow, spleen, liver and brain in NSG mice treated with WT ARI-0001 or TCRKO ARI-0001 cells at the point. **G**) Percentage of T_N_/T_SCM_ +T_CM_ population in hCD2+CAR+ cells analyzed in different mice organs after sacrifice. Only when CAR+ population was >1%, data was included. **(H)** Percentage of hCD3- cells of live cells prior inoculation (*in vitro*) and %hCD3- cells of hCD2+ cells in liver, bone marrow, spleen and brain after sacrifice (*in vivo*). Statistics are based on log rank test **(C)**, unpaired, one-tailed Student´s t-test **(D)**, *p<0.05, **p<0.01. Graphs show mean ± SEM.

We finally compared the anti-tumor efficacy of TCRKO- and WT- ARI-0001 CAR-T cells in a xenograft lymphoma model. Both CAR-T cells were prepared as described previously and frozen in liquid nitrogen (LN). NSG mice were inoculated with Namalwa cells (expressing eGFP-NanoLuc), and three days later, the CAR-T cells were thawed and immediately inoculated intravenously. Disease progression was measured by bioluminescence (**Figure 6A**). Control mice (inoculated with PBS or non-transduced T cells) presented a uniform fast lymphoma progression and were sacrificed 16 days after Namalwa injection. After day 31, mice treated with TCRKO ARI-0001 cells presented 50% survival and mice treated with WT ARI-0001 cells 40% survival. At that point, all surviving mice (including one NT) were re-challenged with Namalwa cells with the objective of determining the efficiency of surviving TCKO TCAR in cured mice. In addition, new PBS controls inoculated with new Namalwa cells were generated and tumor progression was monitored up to day 56 (from the start of the experiment). At this point, both WT and TCRKO ARI-0001 showed a similar survival (20% vs 17%) (**Figures 6B, C**). Of note, the two mice of WT ARI-0001 group were sacrificed as they presented signs of GvHD in the absence of tumor presence, while no signs of xenoGvHD were observed in any mice inoculated with TCRKO ARI-0001 cells (**Figure 6D**).

After sacrifice, we determined the total count of Namalwa cells and CAR T cells in different organs. Tumor cells were completely eliminated by WT ARI-0001 and TCRKO ARI-0001 cells in bone marrow and blood (**Figure 6E**). High levels of CAR T cells were found in all tissues analyzed, with higher expansion in spleen and liver and being the WT ARI-0001 cells more abundant in all tissues, but without statistical significance (**Figure 6F**). Phenotypic analysis of CAR T cells confirmed a similar proportion of T_N_/T_SCM_ +T_CM_ population in both WT and TCRKO ARI-0001 cell treated mice in all the organs analyzed at end point (**Figure 6G**). We did not observe a correlation between the level of infiltration in the different tissues (**Figure 6E**) and the phenotype of CAR T cells. Importantly, the percentage of CD3- cells was comparable to pre-infused T cells in the hematopoietic tissues (**Figure 6H**). Taken together, our studies showed that TCR elimination does not hamper anti-tumoral activity of ARI-0001 cells in a Namalwa xenograft mouse model.

## Discussion

In the present study, we demonstrated the feasibility of generating universal *off-the-shelf* ARI-0001 cells. ARI-0001 is an academic CART19 therapy approved for hospital exemption by the AEMPS for the treatment of B-ALL in patients over 25 years. This autologous CAR-T therapy has reported comparable results with other αCD19 CAR-T products (48) in patients that did not accomplish the requirements for treatment with the available commercial products.

The development of ARI-0001 under hospital exemption certainly offers the opportunity to develop CAR-T therapy in a close contact with clinical practice and for rapid access for patients. However, as mentioned above, patient-specific manufacturing is expensive, time consuming, yield cells product with a heterogeneous potency and is not always possible to generate the final product. The generation of ARI-0001 CAR-T cells using cells from healthy donors would increase the number of patients that could access to the treatment (9). Nonetheless, GvHD risk must be avoided in order for these cells to be safe enough to be considered for clinical use, one of the most efficient strategies relies on eliminating the endogenous expression of αβ T-cell receptor (TCR). Other alternative would be the use of CAR-NK cells or γδ CAR-T cells (49). The fact that NK cell adoptive transfusion has been rarely related with GVHD events (50, 51) and the feasibility of several sources to generate NK cells (52) postulate CAR-NK cells as an interesting alternative for *off-the-shelf* product (49). In the same direction, γδ T cells, another key component of the innate immune system, have also been proposed as an alternative for the generation of allogeneic CAR-T cells (49). In spite of their small starting number in peripheral blood, they can be expanded ex vivo (53, 54) for clinical applications (see (49) for a review). However, there are still several barriers for clinical success of both CAR-NK and γδ CAR-T cells, such as low transduction efficiency and/or limited *in vivo* persistence and expansion (52).

Gene editing has become an exceptional tool for specifically disrupting the expression of a gene of interest for both research and clinical purposes. However, it can also create undesired collateral genotoxicity that needs to be studied in detail. In the case of CRISPR/Cas9 nuclease, the site-specific DNA cleavage generated is mostly repaired by the error-prone NHEJ pathway (55), which commonly generates small indels (< 50pb). Moreover, recent studies have shown a relatively high frequency of large deletions at CRISPR/Cas9 cut sites (31–33, 35), indicating that DNA repair pathways distinct from NHEJ play an important role in DSBs repair. In this study we detected large on-target deletions in the *TRAC* gene ranging 2-3 kb in size, a phenomenon previously unexplored in CAR-T cell field until very recently (33). Around one third of these large deletions contained microhomologies at their ends, suggesting the involvement of microhomology-mediated end joining (MMEJ) in addition to NHEJ as potential mechanism of this large deletions, as it has been previously reported (35, 56). Large deletions can have deleterious effects on nearby genes if they affect regulatory regions. However, the consequences are not restricted to the target locus since these deletions can be important sources of genomic instability. These potential negative effects highlight the importance of extensive genotyping to accurately detect large deletions in cells that intended for treating patients. In recent years, several groups have identified large deletions as a potential genotoxic risk and proposed different alternatives to reduce it. Wen et al. (33) showed that the frequency of large deletions is dependent on the target site and the cell type, human primary T cells being particularly prone to these genotoxic effects. They also showed that genome editing using homology-directed repair (HDR) substantially reduced large deletions. Reducing the number of large deletions is essential to ensure safer T cells editing. TCR editing requires activation, and therefore expansion of T cells, which usually implies a change in the chromatin structure, although in our hand we do not observed significant changes between expanded and no expanded cells. Another alternative to avoid the generation of large deletions is the use of base editors to eliminate the TCR (57).

Despite the potential genotoxic effects of CRISPR/Cas9, we have shown several lines of evidences indicating that TCR disruption does not significantly alter functionality of T cells nor CAR-T cells. Indeed, we have demonstrated minor phenotypic alterations of TCRKO ARI-0001 compared to ARI-0001 CAR-T cells on resting state, after repeated *in vitro* activation as well as in an animal model. In addition, our studies on the mitochondrial metabolism confirmed the absence of mayor effects of the T cell physiology after TCR genome editing.

Remarkably, TCR edition of ARI-0001 T cells completely eliminated allogeneic responsiveness while maintaining a similar *ex vivo* or *in vivo* anti-tumor activity compared to ARI-0001 CAR-T cells. Our data are somehow in contrast to what was described by Stenger et al. work, in which is showed that endogenous TCR promotes *in vivo* persistence of CD19-CAR-T cells (22). However, our results are in line with other works (10, 16) in which survival of Raji- (10) or Namalwa- (16) bearing mice was similar upon treatment with either TCRKO or WT CAR-T cells. Of note, after *in vivo* expansion, WT and TCRKO ARI-0001 cells presented a predominant T_EM_ phenotype, but also a substantial percentage (10-20%) of less differentiated T cells, T_N_/T_SCM_ +T_CM_, which have been correlated with a superior antitumor response in several preclinical and clinical studies (5, 58–60).

In summary, despite the potential risks derived from the generation of large deletions using CRISPR/Cas9, all the data generated in this manuscript indicate a good safe/risk ratio of TCRKO ARI-0001 CAR-T cells with absence of any phenotypic alterations. We therefore propose TCRKO ARI-0001 CAR-T cells generated with CRISPR/Cas9 as a new allogeneic ATMP to be investigated for the *off-the-shelf* treatment of R/R B-cell leukaemias and lymphomas that do not fit the autologous CAR-T requirements.

## Data availability statement

The original contributions presented in the study are included in the article/**Supplementary Material**. Further inquiries can be directed to the corresponding author.

## Ethics statement

The studies involving human participants were reviewed and approved by Comité de etica de la investigación biomedica de la provincia de GRANADA (CEIM/CEI GRANADA). Hospital Universitario San Cecilio. The patients/participants provided their written informed consent to participate in this study. The animal study was reviewed and approved by The Institutional Animal Care and Use Committee of the University of Granada (procedures 92-CEEA-OH2015).

## Author contributions

NM-P and MT-M designed and performed experiments, analyzed data and wrote the manuscript. PJ-L, EM-P, PM, KP, MC-G, and CB-B performed and analyzed experiments. MC and MJ provide material and critical review. MW and PR contributed to metabolic experiments. FM-E, CM, and CH designed experiments and provided critical review. KB designed experiments, analyzed data, provided funding and critical review. FM conceals the project, designed experiments, analyzed data, provided the main funding and wrote the manuscript. All authors contributed to the article and approved the submitted version.

## Funding

This study was funded by the Spanish ISCIII Health Research Fund and the European Regional Development Fund (FEDER) through research grants PI15/02015, PI18/00337, PI21/00298 and Red TerAv RD21/0017/0004 (FM), PI18/00330 (KB) and PI17/00672 (PM); The CECEyU and CSyF of the Junta de Andalucía FEDER/European Cohesion Fund (FSE) for Andalusia provided the following research grants: 2016000073391-TRA, 2016000073332-TRA, PI-57069, PAIDI-Bio326, CARTPI-0001-201, PECART-0031-2020 (FM), PI-0014-2016 (KB) and PEER-0286-2019 (PM); The Ministerio de Ciencia, Innovación y Universidades through research grants 00123009/SNEO-20191072 and PLEC2021-008094 to FM. KB and CM held Nicolas Monardes contracts from regional Ministry of Health (#0006/2018 and C2-0002-2019 respectively). MT-M, NM-P, and AAG are funded by Spanish Ministry of Education and Science through fellowships FPU16/05467, FPU17/02268 and FPU17/04327 respectively. PJ-L and CB-B are funded through industrial doctorate fellowship (MCI DIN2018-010180 and DIN2020-011550 respectively) to LentiStem Biotech. MC-G is funded by grant PECART-0031-2020 from CSyF of the Junta de Andalucia. NM-P, PJ-L, CB-B, and MC-G are PhD students from the Biomedicine Programme of the University of Granada (Spain). PECART-0027-2020 funded by Consejería de Salud y Familias. K.B.

## Acknowledgments

We acknowledge Ana Fernández-Ibáñez for her support with the IVIS Spectrum Analyzer, Nieves Varela Hernández for her help for ELISA assays and Alfredo Caro Maldonado from Agilent Technologies for his assistant with SeaHorse experiments. We also thank GENYO´s supporting units.

## Conflict of interest

Authors MT-M, PJ-L, and CB-B were employed by LentiStem Biotech.

The remaining authors declare that the research was conducted in the absence of any commercial or financial relationships that could be construed as a potential conflict of interest.

## Publisher’s note

All claims expressed in this article are solely those of the authors and do not necessarily represent those of their affiliated organizations, or those of the publisher, the editors and the reviewers. Any product that may be evaluated in this article, or claim that may be made by its manufacturer, is not guaranteed or endorsed by the publisher.
