# Supplementary material for: Efficacy and safety of universal (TCRKO) ARI-0001 CAR-T cells for the treatment of B-cell lymphoma
Source: Front Immunol. 2022 Oct 6;13:1011858. doi: 10.3389/fimmu.2022.1011858 (PMC9585383; doi:10.3389/fimmu.2022.1011858)
Supplement: Supplementary file 1 [file DataSheet_1.pdf]

Suplementary data.

Efficacy and safety of universal (TCRKO) ARI-0001 CAR-T cells for the treatment of type B malignancies.

Noelia Maldonado-Pérez[1†], María Tristán-Manzano[1,2†], Pedro Justicia-Lirio[1,2], Elena Martínez-Planes[1], Pilar Muñoz[1,3], Kristina Pavlovic[1,4], Marina Cortijo-Gutiérrez[1], Carlos Blanco-Beitez[1,2], María Castella[5], Manel Juan[5], Mathias Wenes[6], Pedro Romero[6], Francisco J. Molina-Estévez[1], Concepción Marañón[1], Concha Herrera[4], Karim Benabdellah[1,$] and Francisco Martin[1,7,$,*].

[1]Department of Genomic Medicine, Pfizer-University of Granada-Andalusian Regional Government Centre for Genomics and Oncological Research (GENYO), PTS, Av. de la Ilustración 114, 18016 Granada, Spain.

[2]LentiStem Biotech, Pfizer-University of Granada-Junta de Andalucía Centre for Genomics and Oncological Research (GENYO), PTS, Avda. de la Ilustración 114, 18016 Granada, Spain.

[3]Department of Celular Biology, Faculty of Sciences, University of Granada, Campus Fuentenueva, 18071 Granada, Spain

[4]Maimonides Institute of Biomedical Research in Córdoba (IMIBIC), Cellular Therapy Unit, Reina Sofia University Hospital, University of Córdoba, Córdoba, Spain.

[5]Department of Hematology, ICMHO, Hospital Clínic de Barcelona, Villarroel 170, 08036 Barcelona, Spain.

[6]Department of Oncology, University of Lausanne, Épalinges, Switzerland

[7]Department of Biochemistry and Molecular Biology III and Immunology, Faculty of Medicine, University of Granada, Av. de la Investigación 11, 18007 Granada, Spain.

[†] These authors share first authorship
[$] These authors share senior authorship

*** Correspondence:**
Francisco Martin.
francisco.martin@genyo.es

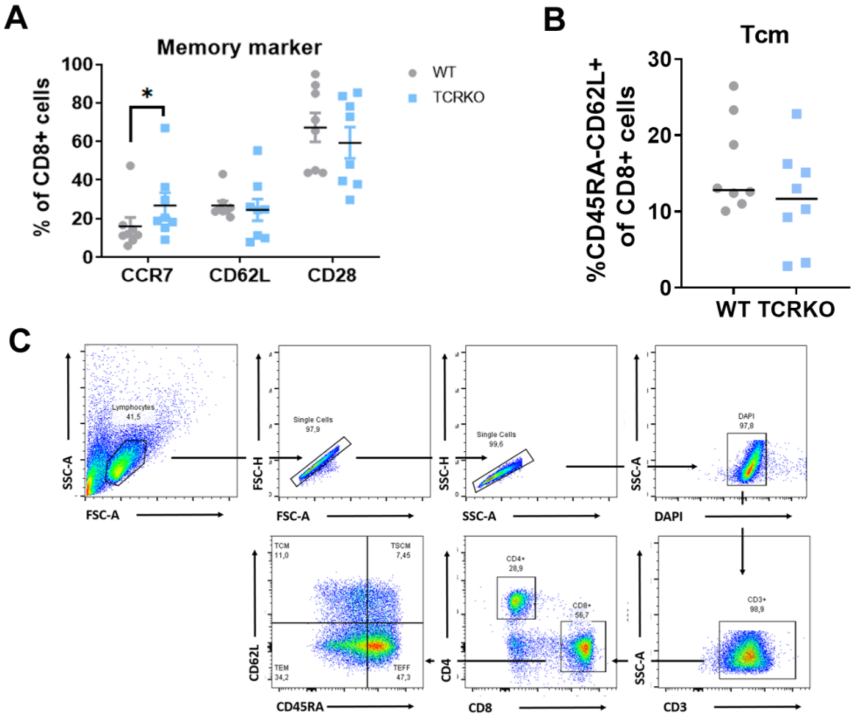

Figure S1. **A**) Graph showing the percentage of CCR7+, CD62L+ and CD28+ in CD8+ T cells, quantified by flow cytometry data at day 9 post activation of TCRKO (Blue) and mock electroporated (WT, gray) T cells. **B**) Graph showing the percentages of T_{CM}, determined as CD45RA-CD62L+, at day 9 post activation of TCRKO (Blue) and mock electroporated (WT, gray) T cells (7 independent donors, n = 8). Statistics are based on paired, two-tailed Student´s t-test (A), *p<0.05. **C**) Gating strategy for T cell phenotype analysis.

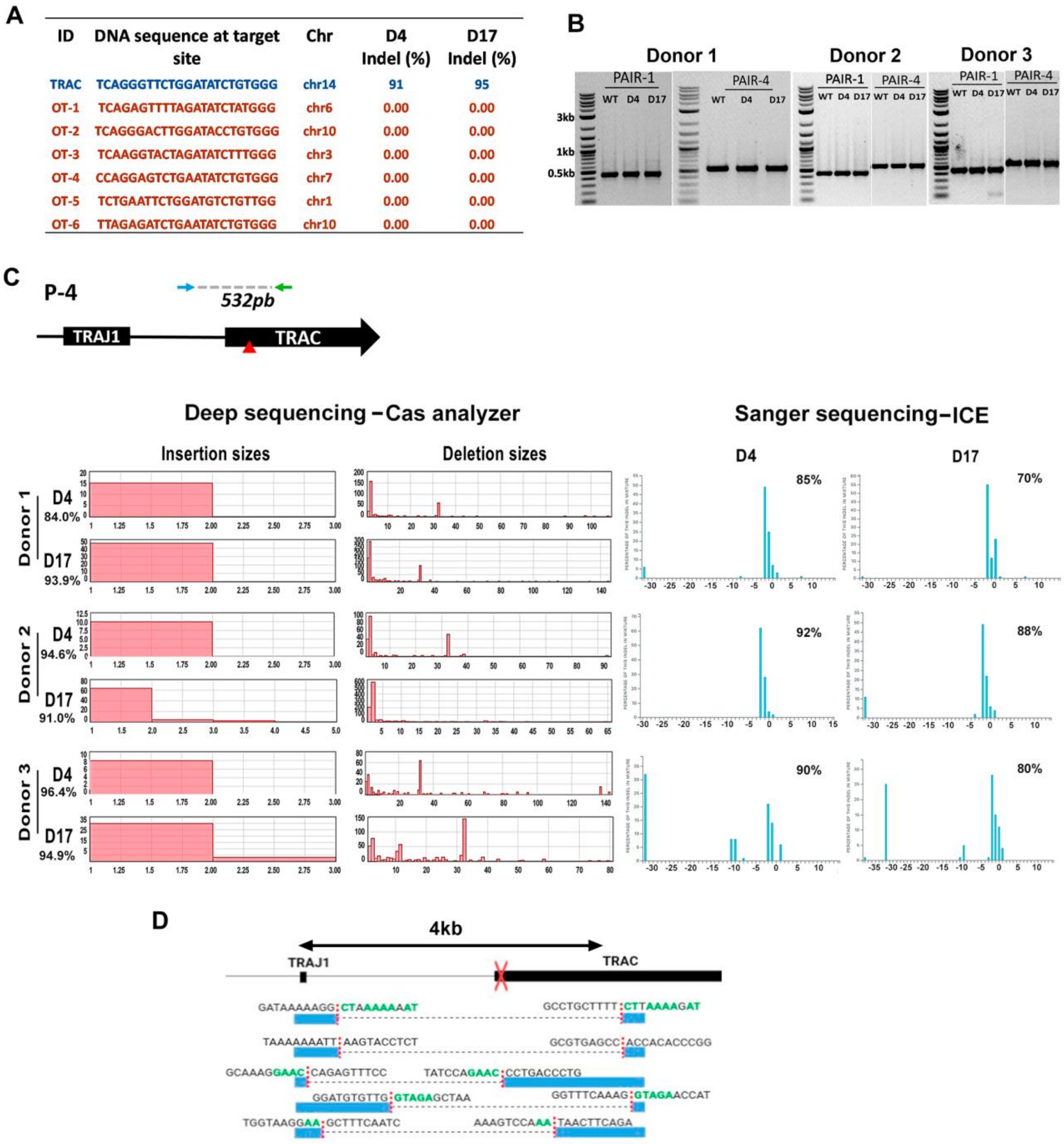

Figure S2. A) Cleavage efficacy (ICE) of gRNA$_{TRAC}$ on predicted 6-top off-target sites at day 4 and 17. **B**) Agarose gels of PCR products obtained after amplification with pair-1 and pair-4 spanning the gRNA$_{TRAC}$ target site of gDNA from non-edited (WT) and edited (D4 and D17) T cells at the different time points. **C**) Top, scheme of the PCR product analyzed after TRAC genome editing using Cas9/ RNP$_{TRAC}$ RNPs. Bottom, analysis of the genome editing efficacy using deep sequencing and Cas analyzer (left) and Sanger sequencing and ICE analysis (right). TCR genome editing was performed in primary T cells from three different donors (indicated at the left) at day 4 and day 17 after isolation. Deep sequence analysis: Indel frequency obtained by Cas analyzer are shown for each day (left-bottom in each day). Insertions (left) and deletions (right) sizes are visualized as graphs. Sanger analysis: Indel frequencies were obtained by ICE analysis and are show at the top-right corner of each graph. Graphs also display the inferred insertions and deletions (indels) distribution. Each bar shows the size of the insertion or deletion along with the percentage of genomes that contains it. **D**) Visual overview of five deletions sequenced and aligned to the reference genome. The top arrow represents the expected amplicon size (4kb). Black boxes represent TRAC and TRAJ1 loci, the Red Cross represent the CRISPR/Cas9 cut site and the red arrows represent the position of the primers used to amplify the deletions. Blue boxes represent the amplicon around the deletion, represented as a dotted line. DNA sequences represent the breakpoints of each deletion and the green bases represent the microhomology matches.

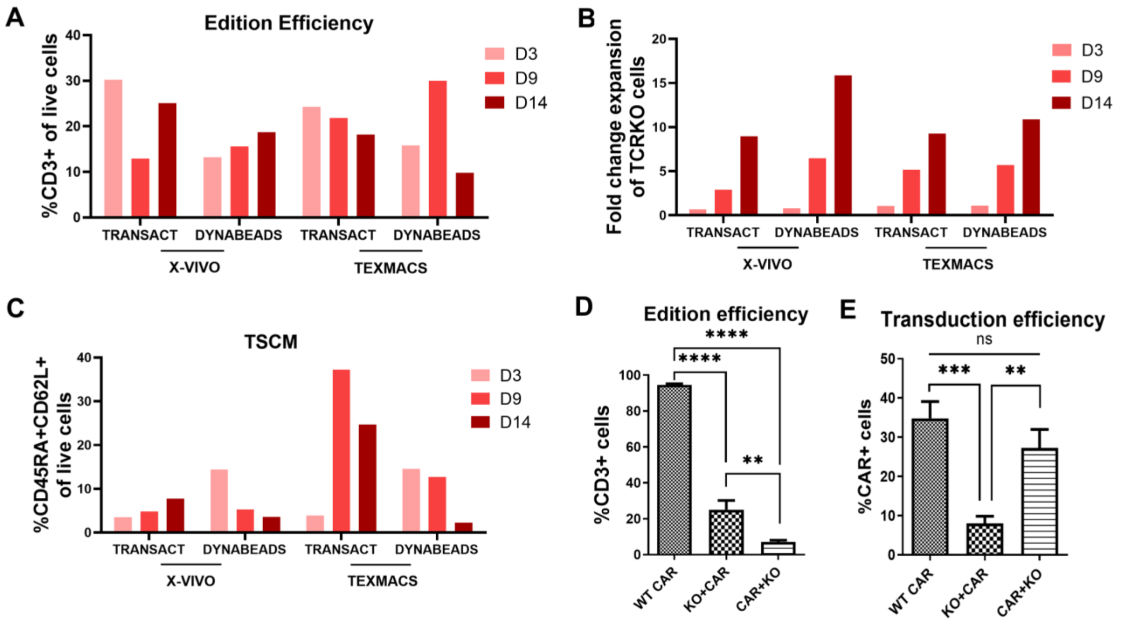

Figure S3. Standardization of the protocol for the generation of TCRKO CAR-T cells. **A**, **B**, **C**) Different media (X-Vivo and TEXMACS) and activation (TRANSACT AND DYNABEADS) protocols were used to determine the best conditions for TCR genome edition using electroporation of the $gRNA_{TRAC}$/Cas9 RNPs. Edition efficacy (**A**), T cell expansion (**B**) and T_{SCM} (CD45RA+CD62L+) (**C**) frequency on edited cells were analyzed for each condition at days 3, 9 and 14 after electroporation. TexMACs or X-VIVO 15 media were supplemented with 20ng/ml IL2 and 5% human serum. **D**, **E**) We studied the best strategy to generate TCRKO CAR T cells. We compared a protocol in which we first eliminated the TCR and then transduced with the LVs expressing the CAR (MOI=10) (KO+CAR) with a protocol in which transduction was performed first (CAR+KO). (**D**) Graph showing edition efficiency of each protocol, analyzed as the percentage of hCD3+ disruption. (**E**) Graph showing the transduction efficiency showed as the percentage of CAR+ cells (2 independent donors, n=5). Statistics are based on paired, two-tailed Student´s t-test, *p<0.05, **p<0.01, ***p<0.005, ****p<0.001 (D, E). Graphs show mean ±SEM.

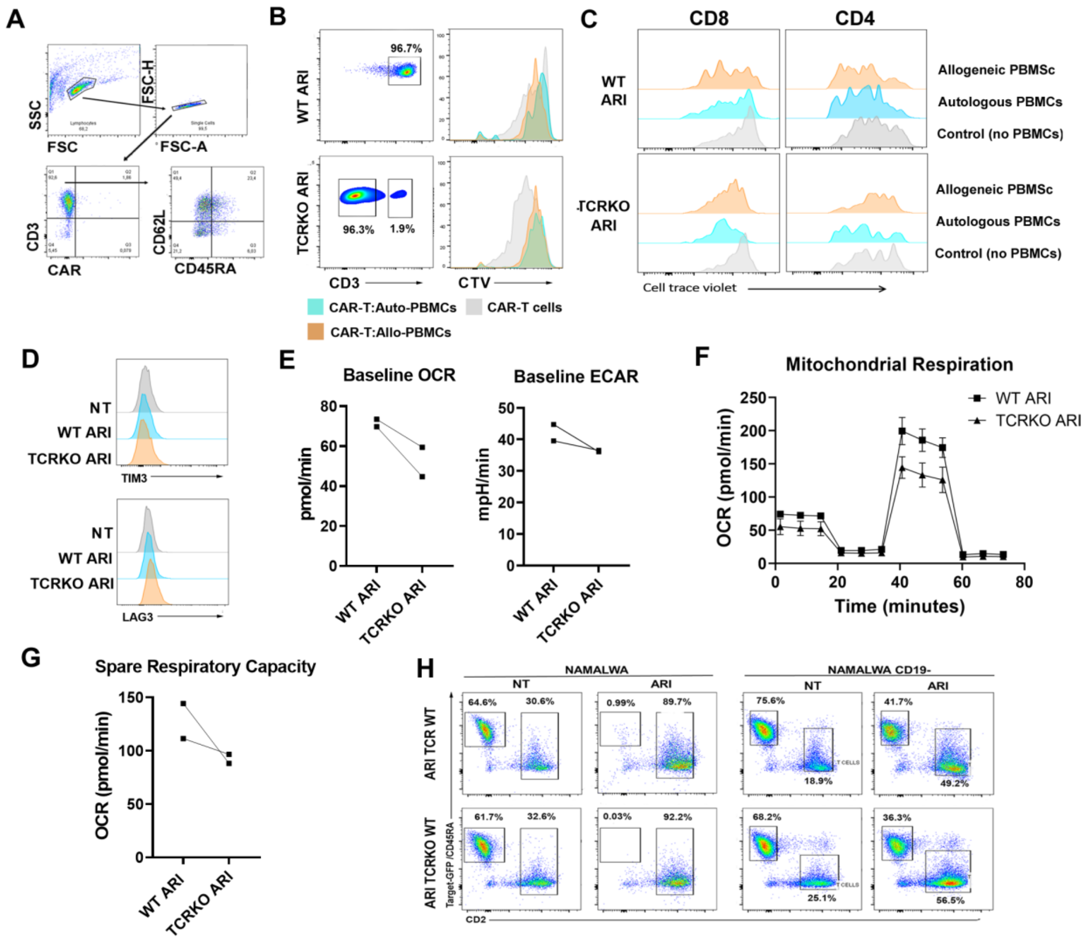

Figure S4. A) Gating strategy for CAR-T cell phenotype analysis. **B**) Panels showing the proliferation of WT (CD3+ top) and TCRKO (CD3- bottom) ARI CAR-T cells in response to allogenic (orange), autologous (blue) PBMCs or non-stimulated CAR-T cells (grey). **C**) Histograms showing the proliferation WT (CD3+ top panels) and TCRKO (CD3- bottom panels) CAR-T cells in response to allogeneic (orange), autologous (blue) PBMCs or non-stimulated CAR-T cells (grey) on CD8 (left panels) and CD4 (Right panels). **D**) Histograms of TIM3 (top) and LAG3 (bottom) expression in NT, WT ARI-0001 and TCRKO ARI-0001 cells. **E**) Graphs showing Mean basal oxygen consumption rate (OCR)(left) and extracellular acidification rate (ECAR)(right). **F**) Graph showing mitochondrial respiration traces (oxygen consumption rate (OCR)) (**G**) Graph showing Spare Respiratory capacity (maximal OCR - basal OCR). Data for graphs E, F and G were obtained using the T cell metabolic profiling kit (Agilent technologies) Donors =2. Three replicates each. **H**) Representative dot-plots showing lytic activity of WT ARI-0001 and TCRKO ARI-0001 against Namalwa (GFP+, left) and Namalwa CD19- (GFP+, right). Percentage of GFP+ cells (target cells) and percentage of CD2+ cells (T cells) are indicated.

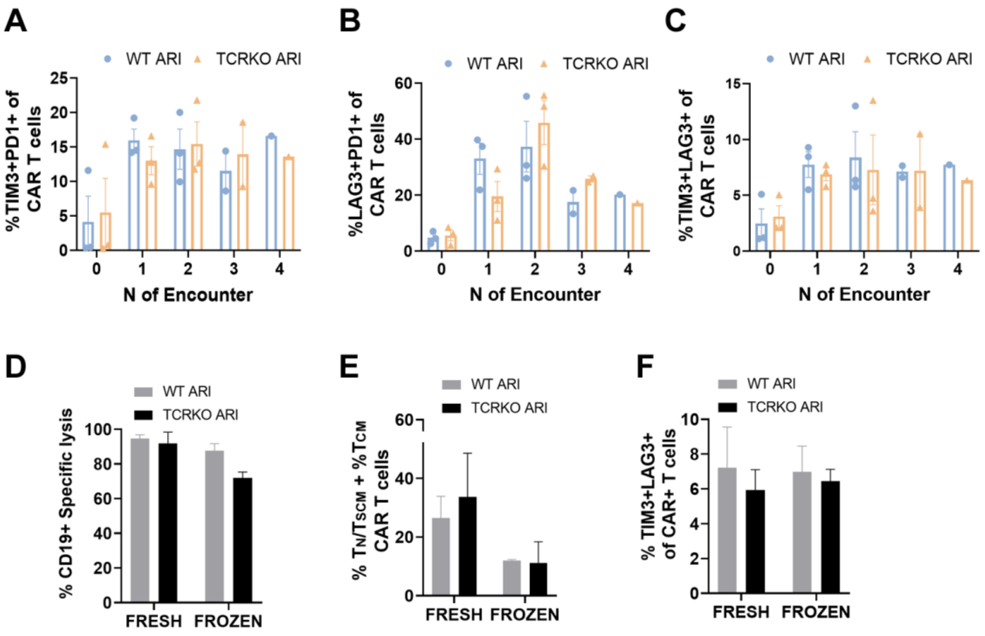

Figure S5. A) Graph displaying the percentage of TIM3+PD1+ **B**) LAG3+PD1+ and **C**) TIM3+LAG3+ of CAR+ T cells before and after different encounters with Namalwa cells (4 independent donors, n=4). **D**) CD19+ specific lytic activity of fresh (left) or frozen (right) WT (gray) and TCRKO (black) ARI-0001 cells. **E**) Percentage of memory T cells (T_{SCM}+T_{CM}) and **F**) percentage of TIM3+LAG3+ after the first encounter with target cells of fresh (left) or frozen (right) WT ARI-0001 (gray) and TCRKO (Black) ARI-0001 cells (2 independent donors, n=2). Graphs show mean ±SEM.

Table S1

ID	Forward (5´to 3´)	Reverse (5´to 3´)
**PAIR-1**	TTGATAGCTTGTGCCTGTCC	GAATAATGCTGTTGTTGAAGGC
**PAIR-4**	TCTGCCAGAGTTATATTGCTG	TTGAAGTCCATAGACCTCATGTC
**PAIR-8**	CTCTGAGAGGGGTCATCCCA	GGCAGCGAGGCATACATAGT
**OT-1**	AGCAGACATTTGGTTTGTGGG	AGCTGATACCTTGCACTCTCC
**OT-2**	GCACTTTCCAATGTGGCCG	ACAGCCTGCCTCAATGTTGT
**OT-3**	CTCTGAATCCCACAACCCTGA	AGCCATCCTTCCCATCTGTT
**OT-4**	TCCACAACGGATTTGTTCCAG	CCAGCTTACTGCCTGAGGT
**OT-5**	ACCCATGCAATGTGCTTAACAT	CTCCCCCTGCTATGTGTTCA
**OT-6**	GACCAATACAGGGGCTGCTT	CCATTTGCATGCACAACCCA

Table S1. Description and sequence of primers used in this study.
